# The Genes of Freedom: Genome-Wide Insights into Marronage, Admixture and Ethnogenesis in the Gulf of Guinea

**DOI:** 10.3390/genes12060833

**Published:** 2021-05-28

**Authors:** João Almeida, Anne-Maria Fehn, Margarida Ferreira, Teresa Machado, Tjerk Hagemeijer, Jorge Rocha, Magdalena Gayà-Vidal

**Affiliations:** 1 CIBIO-Centro de Investigação em Biodiversidade e Recursos Genéticos, Universidade do Porto, 4485-661 Vairão, Portugal; jotabrochado@gmail.com (J.A.); afehn@cibio.up.pt (A.-M.F.); margaridamccferreira@gmail.com (M.F.); maria.teresa.viana.machado@gmail.com (T.M.); magdagaya@gmail.com (M.G.-V.); 2 CIIMAR/CIMAR—Interdisciplinary Centre of Marine and Environmental Research, University of Porto, 4450-208 Matosinhos, Portugal; 3 Department of Linguistic and Cultural Evolution, Max-Planck Institute for the Science of Human History, 07745 Jena, Germany; 4 Department of Medical Sciences, Institute of Biomedicine—iBiMED, University of Aveiro, 3810-193 Aveiro, Portugal; 5 Centro de Linguística da Universidade de Lisboa, 1600-214 Lisboa, Portugal; t.hagemeijer@letras.ulisboa.pt; 6 Faculdade de Letras, Universidade de Lisboa, 1600-214 Lisboa, Portugal; 7 Departamento de Biologia, Faculdade de Ciências, Universidade do Porto, 4169-007 Porto, Portugal

**Keywords:** slave trade, social selection, expanded exome sequences, WES, São Tomé and Príncipe, African populations

## Abstract

The forced migration of millions of Africans during the Atlantic Slave Trade led to the emergence of new genetic and linguistic identities, thereby providing a unique opportunity to study the mechanisms giving rise to human biological and cultural variation. Here we focus on the archipelago of São Tomé and Príncipe in the Gulf of Guinea, which hosted one of the earliest plantation societies relying exclusively on slave labor. We analyze the genetic variation in 25 individuals from three communities who speak distinct creole languages (Forros, Principenses and Angolares), using genomic data from expanded exomes in combination with a contextual dataset from Europe and Africa, including newly generated data from 28 Bantu speakers from Angola. Our findings show that while all islanders display mixed contributions from the Gulf of Guinea and Angola, the Angolares are characterized by extreme genetic differentiation and inbreeding, consistent with an admixed maroon isolate. In line with a more prominent Bantu contribution to their creole language, we additionally found that a previously reported high-frequency Y-chromosome haplotype in the Angolares has a likely Angolan origin, suggesting that their genetic, linguistic and social characteristics were influenced by a small group of dominant men who achieved disproportionate reproductive success.

## 1. Introduction

**Figure 1:**
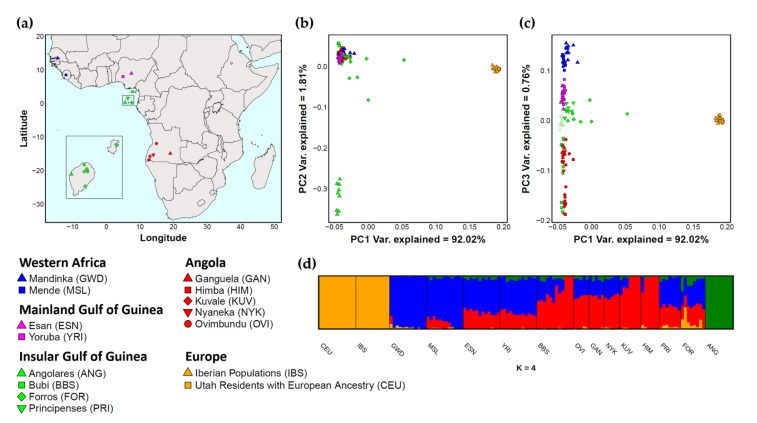
Genetic structure in groups from São Tomé and Príncipe in relation to European and mainland African populations. (**a**) Geographic locations of sampled individuals from São Tomé and Príncipe, and populations on the African continent. In the inset, the distance between São Tomé (larger island) and Príncipe (smaller island) is not to scale. (**b**,**c**) Haplotype-based principal component analysis performed with CHROMOPAINTER/fineSTRUCTURE; (**b**) PC1 and PC2 plot; (**c**) PC1 and PC3 plot. (**d**) ADMIXTURE analysis assuming 4 clusters (K). Each individual is represented as a vertical line divided according to the proportion of its genome that is derived from the assumed genetic clusters. Although the lowest cross-validation error (CV) was associated with K = 2, the differentiation between African mainland populations is evident only from K = 4. Additional PCA and ADMIXTURE plots are shown in Supplementary Figures S2 and S3.

During the first half of the 16th century, the archipelago of São Tomé and Príncipe, located in the Gulf of Guinea (1° N, 7° E), became one of the first examples of the so-called “plantation complex”, which was soon to take over the New World [1] (Figure 1a). When the Portuguese reached the Gulf of Guinea in the early 1470s, they found the islands of São Tomé (860 km^2^), Príncipe (136 km^2^) and Annobón (17 km^2^) to be uninhabited. A fourth island, Fernando Pó (now Bioko), located only 32 km off the coast of Cameroon, had already been populated by the Bubi, an autochthonous Bantu-speaking group who had presumably reached its shores by canoe [2]. While São Tomé and Príncipe were both settled in the last decade of the 15th century, the permanent settlement of Annobón only started in the mid-16th century, and the island always remained sparsely populated until it was ceded to Spain, together with Bioko, in 1778. During their intertwined history, the islands of São Tomé and Príncipe saw the rise of sugar cane cultivation, African slave labor and marronage, which led to the emergence of new ethnic identities and new languages.

After a short homestead phase in which slaves from the African mainland were predominantly imported as domestic servants, the large-scale production of sugar soon required a substantial labor force, which was first acquired through trade with the Kingdom of Benin (in present-day Nigeria) and later with regions in modern-day Democratic Republic of Congo and Angola. In addition to the local work force, São Tomé and Príncipe also served as entrepôts for slaves meant for re-export to the Americas, in particular to the Spanish Main and Brazil [3,4,5]. When slave rebellions and competition with Brazil caused the sugar cane-based economy to collapse by the end of the 16th century, the islands became a provider of fresh water and supplies, and kept a small role as an entrepôt in the Atlantic slave trade [5,6]. Only in the 19th century, after slavery had been abolished, the introduction of coffee and cacao reawakened interest in the archipelago and led to a substantial influx of indentured laborers from Cape Verde, Angola and Mozambique [5,7].

The cultural interactions that characterized the peopling of São Tomé and Príncipe led to the emergence of three distinct Portuguese-related creole languages: (1) Santome (also Lungwa Santome) is spoken by about 60,000 of São Tomé’s 180,000 inhabitants who identify themselves as Forros (literally “free slaves”), as they mostly descend from slaves that were granted manumission throughout the history of the island [7]; (2) Angolar (also Lunga Ngola) is spoken by approximately 10,000 individuals in the northwestern and southeastern parts of the island of São Tomé by the so-called Angolares, the descendants of a self-governed community whose exact origin remains unknown [4,7,8]; (3) Principense (also Lung’Ie) is a severely endangered language that is presently spoken by less than 100 of Príncipe’s 7000 residents. Lung’Ie speakers, also known as Principenses, trace their roots to slaves brought to the island [9]. A fourth Portuguese-related creole language (Fa d’Ambô) originating in Annobón has approximately 5000 speakers [10].

Despite the lack of mutual intelligibility, all these languages can be traced to a single Gulf of Guinea proto-creole that likely emerged in São Tomé during the short homestead period in the early 16th century [11]. Due to the intensified contact between Portuguese colonizers and slaves from the Kingdom of Benin, the new language was characterized by a predominantly Portuguese-based lexicon and a predominantly Nigerian, Edoid-related syntax [11,12]. With the quickly increasing number of Bantu-speaking slaves from Congo and Angola after 1510/1520 [13], Bantu features from Kikongo (from Congo and Angola) and Kimbundu (from Angola) made their way into the lexicon and phonology of the proto-creole [11]. However, since the proto-language had already started stabilizing, these features can be interpreted as adstratum or secondary contact, i.e., borrowings which entered the language in the lexical domain [11]. Among the modern languages, the Bantu element is almost absent in Lung’Ie on the island of Príncipe, which remained less affected by the influx of the Bantu labor force [9,11]. Conversely, Lunga Ngola displays substantial lexical influence from Kimbundu, including in the core lexicon, as well as phonological traits related to Bantu [11,14,15], while Lungwa Santome occupies an intermediate position with lexical contributions from Kikongo and, to a lesser extent, Kimbundu [11,16].

The comparatively strong lexical Bantu contribution from especially Kimbundu in the language of the Angolares appears to be in line with their ethnic designation [9,14,15]. There is, however, an ongoing debate on the origins and history of the Angolar community [4]. While claims about an autochthonous origin [17], similar to that of the Bubis from the neighboring island of Bioko, seem unsubstantiated and have received relatively little attention [4], two main hypotheses about the foundation of the Angolar community are still being debated. The first dates back to the first half of the 18th century and considers the Angolares to be the survivors of the wreckage of a slave ship from Angola at a group of rocky islets located 5 km from the southern coast [4,8]. Interestingly, this scenario, which plays an important role in the Angolar folklore [9], bears striking similarities to the traditional views on the origins of the Garifuna from St. Vincent Island in the Caribbean [18,19]. However, as it has been impossible to identify the date or location of the shipwreck, some historians speculate that the story may have been circulated to disguise the vast number of slave escapes from the plantations themselves [20]. The second and most widely accepted hypothesis assumes that the Angolares are in fact the descendants of a maroon community formed by slaves who managed to escape to the forest after they had been brought to the island as plantation workers or for re-export to the Americas [4,8,21]. Slave rebellions and escapes to the mountainous regions of São Tomé date back to the first days of colonization during the late 15th and early 16th century, culminating in a major uprising in 1595 [4]. While the existing sources do not document a clear link between these events and the modern Angolar community [4], they do show that no shipwreck scenario was needed for the formation of maroon communities on the island.

We have previously detected an unusually strong signal of genetic differentiation between the Angolares and the remaining populations of the island of São Tomé by using a set of just 15 autosomal microsatellite polymorphisms [22]. However, this extreme differentiation did not allow us to recover the historical relationships between the Angolares and other groups from São Tomé and Príncipe and from the African mainland. 

Here, we reassess the genetic variation of the three speech communities of São Tomé and Príncipe (Forros, Principenses and Angolares) using genomic data from expanded exomes in combination with a contextual dataset from Europe and from major slave-trading zones in Africa, including new data from Angola. We found that, despite the strong levels of differentiation of the Angolares, the three groups share notable genetic similarities with respect to their Gulf of Guinea/Angolan ancestry ratios, suggesting that the Angolares are an admixed isolate. Based on the available genetic and linguistic evidence, we further propose that their origins trace back to a maroon community strongly influenced by the political and cultural dominance of one or several related men from Angola.

## 2. Materials and Methods

### 2.1. Population Samples

We generated 53 expanded exomes from 9 Angolares (ANG), 8 Forros (FOR) and 8 Principenses (PRI) from São Tomé and Príncipe, as well as from 28 individuals belonging to five Angolan populations: 5 Ovimbundu (OVI), 5 Ganguela (GAN), 5 Nyaneka (NYK), 6 Himba (HIM) and 7 Kuvale (KUV). Samples from Forros and Angolares were obtained from Lungwa Santome and Lunga Ngola speakers whose four grandparents were born in villages where the two languages are still used as medium of everyday conversation. Although Lung’Ie is highly endangered, we could sample five individuals who still were active speakers and three additional individuals who only had a passive knowledge of the language. In all cases, all four grandparents were speakers of Lung’Ie and had been born on the island of Príncipe. Additional details on sampling procedures in São Tomé and Angola have been described elsewhere [22,23,24] (Figure 1a; Supplementary Table S1). 

### 2.2. Expanded Exome Sequencing, Variant Calling and Quality Control

DNA samples were extracted from buccal swabs and saliva as previously described [24,25]. Library preparation for expanded exome sequencing (~62 Mb) was done using the Nextera^®^ Rapid Capture Enrichment kit by Illumina, San Diego, CA, USA, following the protocol version #15037436 v01. Indexed samples were sequenced in two runs on an Illumina’s HiSeq 1500 System with 250 cycles in paired-end mode. A mean depth coverage of 21x (5-41x) was obtained for captured regions. The 53 newly generated exomes were compared with sequence data from 71 individuals with average genome coverage 13x, belonging to six reference populations from the 1000 Genomes Project [26] (ftp://ftp.1000genomes.ebi.ac.uk/vol1/ftp/phase3/data/ accessed on 21 October 2017), as well as with previously reported data from 12 Bubi (BBS) individuals (coverage 27x) from the island of Bioko [2] (https://www.ebi.ac.uk/ena/browser/view/PRJEB26599 accessed on 31 July 2019) (Supplementary Table S1). To avoid ascertainment bias, we reanalyzed the raw sequence data from these populations together with the newly generated data, instead of just merging the different datasets. Due to lack of available genome-wide data, Fa d’Ambô speakers from the island of Annobón were not considered in the present study. 

We performed a quality control check with FastQC (v0.10.1) [27] and applied a filter for Phred Quality Score of 30 (Q30) using Sickle (v1.33) [28] in pair-end mode. Quality reads were aligned to the reference genome GRCh37/b37 using the -mem option of the Burrows-Wheeler Aligner (BWA) software (v0.7.15) [29]. File conversion, sorting, indexing and merging were done with SAMtools (v1.3.1) [30,31]. PCR duplicate reads were flagged with the MarkDuplicates tool from the Picard toolkit (v2.8.0) (http://broadinstitute.github.io/picard accessed on 15 December 2016). Variant discovery workflow was done with the Genome Analysis Tool Kit (GATK) v3.4.46, following GATK Best-Practices recommendations for exome sequencing [32,33,34]. When recommended, the enrichment captured regions were analyzed with additional 100 bp of padding. After obtaining a variant calling file (vcf) with GATK HaplotypeCaller for each individual, we ran the joint genotyping tool GATK GenotypeGVCFs. Next, we performed variant quality score recalibration (VQSR) as recommended, using a sensitivity threshold of 99% for both SNPs and insertions/deletions (Indels). The number of variants that passed this filter was 799,704.

To improve the quality of the variant dataset, we further filtered the data with VCFtools (v0.1.13) [35], retaining only autosomal biallelic SNPs (average coverage 16x) without excessive coverage (<35x). Moreover, a minimum genotype coverage of 3 and a minimum genotype quality of 20 were required; with these filters, sites with >15% missing data were excluded. Sites with a Hardy–Weinberg equilibrium *p*-value < 0.05 for at least two populations were also excluded. Overall, the filtering process yielded 149,501 autosomal SNPs. 

All individuals were checked for relatedness with the -relatedness2 option on VCFtools (v0.1.13) [36,37]. One individual from a pair of samples from Príncipe with a kinship coefficient of 0.248 (first-degree) was removed. The final dataset consisted of 135 samples from 15 populations with 149,501 SNPs with a transition/transversion ratio (Ti/Tv) of 2.58, confirming the high quality of the sequences.

### 2.3. Population Structure Analyses

**Figure 2:**
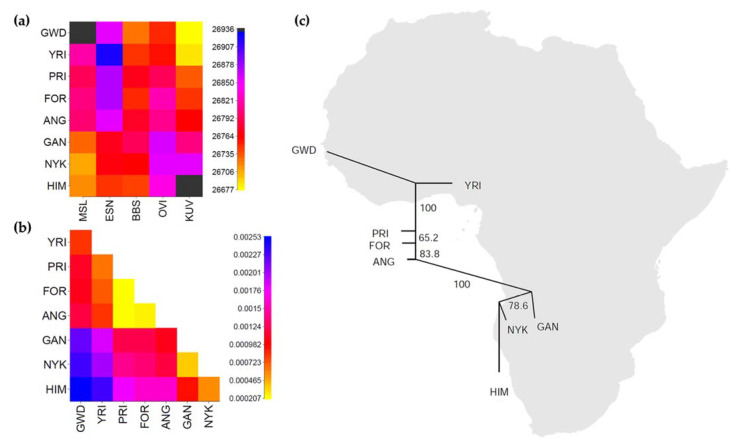
Ancestry inference in the studied populations. (**a**) CHROMOPAINTER coancestry matrix based on the number of haplotype segments (chromosome chunks) shared between donor (columns) and recipient (rows) populations. The copy profile of each recipient group is an average of the copy profiles of all individuals belonging to that group. (**b**) Matrix of pairwise TVDxy distances based on the ancestral profiles of the recipient groups in panel (**a**). The scales of chunk counts and TVDxy values are shown to the right of the matrices. (**c**) Neighbor-Joining (NJ) tree based on TVDxy distances. Values indicate the percentage of NJ partitions observed in 1000 replicas of the coancestry matrix, generated by sampling the individual copy profiles from each population with replacement. The NJ tree was rotated to fit the approximate geographic location of the recipient groups. Abbreviations: GWD (Mandinka), MSL (Mende), ESN (Esan), YRI (Yoruba), ANG (Angolares), BBS (Bubi), FOR (Forros), PRI (Principenses), GAN (Ganguela), HIM (Himba), KUV (Kuvale), NYK (Nyaneka), OVI (Ovimbundu).

Haplotype-based coancestry matrices, principal component analysis (PCA) and clustering dendrograms were obtained with fineSTRUCTURE/CHROMOPAINTER v.2 [38]. Phasing and genotype imputation were carried out with BEAGLE (v4.1) [39,40]. We calculated two types of coancestry matrices. In the first type, we assumed that the haploid genomes of each individual are formed by copying DNA chunks from any other individual in the whole sample, independently of the group to which that individual belongs (Supplementary Figure S4a). In the second type of matrix, we defined a group of recipients whose haploid genomes were copied from a group of donors belonging to a specific set of populations (Figure S2a). The differences between the average copy profiles of pairs of recipient populations were quantified using the total variation distance (TVDxy) [41,42] and visualized with a Neighbor-Joining (NJ) consensus tree with weighted branches using SplitsTree4 [43] (Figure 2c). Support for NJ partitions was calculated by generating 1000 replicas of the original coancestry matrix by sampling with replacement the copy profiles of individuals from each recipient population.

To estimate mutation emission and recombination scaling parameters used in the analyses relying on CHROMOPAINTER, we performed initial runs using 10 iterations of the Expectation-Maximization (EM) algorithm for a subset of five randomly selected chromosomes (chr1, chr6, chr11, chr16 and chr21). The inferred parameters were first averaged by chromosome (weighted by their number of SNPs) and then by individuals. These parameters were then used in subsequent CHROMOPAINTER runs on all individuals and chromosomes. 

Genotype-based, unsupervised clustering analyses were performed by applying ADMIXTURE v1.3.0 [44] to a linkage disequilibrium (LD) pruned dataset consisting of 62,564 SNPs, obtained with the PLINK –indep-pairwise option [45], using a 200-SNP sliding window incremented by 5 SNPs, and a LD threshold of r^2^=0.2. We performed 20 independent ADMIXTURE runs for each K value from 2 to 5, applying a cross-validation (CV) procedure. The results were post-processed and plotted with the pong software [46].

Pairwise Fst values between populations were calculated with EIGENSOFT [47,48] and visualized with a heatmap and UPGMA clustering with the Pheatmap package [49].

We used PCA (Figure 1c) and ADMIXTURE (K = 4) (Figure 1d) to quantify European, Gulf of Guinea and Angolan ancestral contributions to the Forros and Principenses. Using ADMIXTURE, the European contribution was simply taken as the proportion of the European ancestry component (orange) in each population. To estimate the Gulf of Guinea and Angolan contributions, we calculated the average proportions of the ancestry components in blue and red in Gulf of Guinea (Esan and Yoruba) and Angolan (Ovimbundu, Ganguela and Nyaneka) populations, and we used these proportions in Bernstein’s equation [50]. All three parental groups (Europe, Gulf of Guinea, Angola) consist of individuals that were assembled into homogeneous clusters using fineSTRUCTURE. The pastoralist Kuvale and Himba form a distinct subcluster in Angola and were not considered in this analysis (Supplementary Figure S4b).

For PCA, we determined the centroids of the European, African, Gulf of Guinea and Angolan populations in PC1 + PC3. To obtain the European admixture proportions we projected each Forro and Principense individual onto the line connecting the European and African centroids and estimated the European contribution as one minus the Euclidian distance of each projection to the European centroid, divided by the Euclidian distance between the European and the African centroids. We repeated the same procedure to obtain the contributions from the Gulf of Guinea using the line connecting the Gulf of Guinea and Angolan centroids [51,52]. Since the Gulf of Guinea/Angolan ancestry proportions of the Angolares could not be assessed with these methods, we additionally used an ad hoc approach based on the relative positions of populations in the NJ tree obtained from the TVDxy distances (Figure 2c). In this approach, the Gulf of Guinea contribution was given by the Euclidean distance between the root of each population from São Tomé and Príncipe to the Yoruba, divided by the distance between the Yoruba and the midpoint between the roots of the Ganguela and Nyaneka.

### 2.4. Genetic Diversity

We characterized genetic diversity using observed, individual per locus heterozygosities (Ho), runs of homozygosity (ROH), LD measured by the squared correlation of allele frequencies (r^2^), folded Site Frequency Spectra (SFS) and individual inbreeding coefficients (Fis). To control for uneven sample sizes in LD, SFS and Fis calculations, we downsampled the number of individuals to 5 (the sample size of the Ovimbundu, Nyaneka and Ganguela). We repeated this process ten times, calculating each summary statistic in each replicate, and taking the average over replicates as the final estimate.

ROH with a minimum length of 500 kb were calculated with PLINK 1.9 with options: -homozyg-density 50, -homozyg-gap 500, -homozyg-kb 500, -homozyg-snp 100, -homozyg-window-het 1, -homozyg-window-missing 5, -homozyg-window-snp 50 and -homozyg-window-threshold 0.05. We report the total number of ROH segments (nROH) and the total length of ROH (sROH).

We calculated LD (r^2^) between pairs of SNPs in sliding windows of 1 Mb in each population using PLINK 1.9. To evaluate the LD decay, we binned the LD values between pairs of SNPs according to different genomic distance categories (<2 Kb, 2–5 Kb, 5–10 Kb, 10–15 Kb, 15–20 Kb, 20–25 Kb, 25–30 Kb, 30–35 Kb, 35–40 Kb and >45 Kb) and calculated the mean r^2^ value within each bin.

The SFS and Fis statistics were also calculated with PLINK, using —freq and —het, respectively. Ho was calculated by dividing the number of polymorphic sites in each individual by the total number of SNPs in the dataset.

### 2.5. Mitochondrial DNA and Y-Chromosome Variation

We compared newly generated data from the island of Príncipe consisting of mitochondrial DNA (mtDNA) sequences of hypervariable regions (HVR) I and II, and Y-chromosome microsatellite haplotypes, with previously reported data from the island of São Tomé [22]. MtDNA sequences and haplotypes defined by 11 Y-chromosome microsatellite loci (Powerplex Y system, Promega) were obtained for 41 maternally unrelated and 19 paternally unrelated individuals, carrying lineages that could be associated with at least one Lung’Ie speaker up to the grandparental generation. MtDNA sequencing and Y-chromosome typing were done as described [22]. MtDNA haplogroups were assigned with HaploGrep [53]. Y-chromosome haplogroups were inferred from microsatellite haplotypes with Haplogroup Predictor (http://www.hprg.com/hapest5/ accessed on 24 May 2020) [54]. 

Haplotype networks were built with the NETWORK 10.2 software (Fluxus Technology Ltd., Sudbury, UK, http://www.fluxus-engineering.com, accessed on 28 December 2020) using the median-joining algorithm alone (mtDNA) or in combination with a reduced-median algorithm (Y-chromosome) [55,56]. Molecular diversity indices were calculated with the ARLEQUIN 3.5.2.2 software [57].

The TMRCA for a previously identified [22] Y-chromosome descent cluster reaching high frequencies in the Angolares was calculated with the rho statistic [58,59], using an average microsatellite mutation rate of 0.0025 per locus per generation [60,61] and a generation time of 30 years [62].

## 3. Results

### 3.1. Genetic Structure

Using newly generated expanded exome data, we compared the genetic composition of three creole-speaking communities from São Tomé and Príncipe with five populations from Angola, as well as with available sequence data from Europe and from major slave-trading regions in Africa (Figure 1a; Supplementary Table S1).

In a haplotype-based principal component analysis (PCA) implemented by the fineSTRUCTURE algorithm [38], the Angolares show no detectable European ancestry (PC1) and are clearly separated from all other African populations (PC2) (Figure 1b). A pairwise Fst analysis further shows that the average genetic distances between the Angolares and their neighbors from São Tomé and Príncipe (Fst = 0.027) are as high as their distance to other African populations (Fst = 0.028) (Supplementary Figure S1). This differentiation is also confirmed in a LD pruned dataset using ADMIXTURE [44] (Figure 1d).

Consideration of additional PCs (Figure 1c; Supplementary Figure S2) reveals a north–south gradient of relationship across different geographic and linguistic regions of Africa (PC3), with a maximum divergence between the Mande-speaking Mandinka from Gambia and the Bantu-speaking Himba and Kuvale from southwestern Angola. This gradient is further supported by the ADMIXTURE results, which show a southward decrease in a genetic component associated with Mande-speakers (blue) accompanied by an increase in a genetic component that predominates among Bantu-speaking groups (red) (Figure 1d; Supplementary Figure S3).

While the position of the divergent Angolares cannot be determined in this genetic gradient, the Forros and Principenses lie between the Esan and Yoruba from Nigeria, and a group of Bantu-speakers from Angola that includes the Nyaneka, the Ganguela and the Ovimbundu (Figure 1c). This observation is compatible with the available historical records, which identify Nigeria/Gulf of Guinea and Congo/Angola as the two most relevant slave-trading areas involved in the settlement of São Tomé and Príncipe [11].

In contrast to the Forros and Principenses, the Bubi from the neighboring island of Bioko are grouped together with Bantu-speaking populations (Figure 1c). The Bubi sample, however, is quite heterogeneous, and three individuals overlapping in PC3 with the Kuvale and Himba are in fact separated as extreme outliers by PC4 (Figure 1c; Supplementary Figure S2); these individuals, together with three additional samples showing less extreme differentiation, have previously been grouped in a distinct genetic cluster using whole genome data [2]. 

To better elucidate the relationship between the Angolares and other groups, we tried to reduce the impact of their genetic differentiation by further exploring haplotype sharing profiles generated by CHROMOPAINTER [38,42]. In this analysis (Figure 2a), we split the African populations into a group of donors and a group of recipients, assuming that the haplotypes of recipients were exclusively formed by DNA chunks from donor populations. The recipient group included the three language communities from São Tomé and Príncipe as well as populations from West Africa (Mandinka), Gulf of Guinea (Yoruba) and Angola (Ganguela, Nyaneka, Himba). The donor group consisted of the remaining populations, all from geographical areas located as close as possible to the recipients: Mende in western Africa; Esan in the Gulf of Guinea; Ovimbundu and Kuvale in Angola. To account for a possible contribution of Bioko to São Tomé and Príncipe, the Bubi were also included in the donor group.

Figure 2a presents a coancestry matrix based on the number of haplotype segments shared between donors and recipients. As expected, the haplotype copy profiles show that recipient populations from the African mainland derive most of their haplotypes from donor groups that match their geographic and linguistic area. Figure 2b displays pairwise total variation distances (TVDxy) between recipients, calculated on the basis of their inferred African ancestries [38,42]. Remarkably, when genetic similarity is assessed only on the basis of ancestry, the three groups from São Tomé and Príncipe become very close to each other, suggesting that the genetic uniqueness of the Angolares was caused by demographic events occurring within the island of São Tomé, rather than by different external contributions from the African mainland (Figure 2b). The genetic similarity between Angolares, Forros and Principenses is further illustrated by a Neighbor-Joining network calculated with the TVDxy matrix, which, as expected, is closely related to geography (Figure 2c).

We additionally used both PCA and ADMIXTURE to quantify the ancestral contributions of Europe (Iberians and CEU-Europeans), Gulf of Guinea/Nigeria (Esan and Yoruba) and Congo/Angola (Ganguela, Nyaneka and Ovimbundu) to the genome of Forros and Principenses (Supplementary Table S2, Supplementary Figure S4). Although the Ovimbundu, Ganguela and Nyaneka are located to the south of more relevant slave trade areas from Angola, these populations can still be considered adequate proxies for the Congo/Angola ancestry, as they have been shown to be genetically very similar to the Kikongo and Kimbundu-speaking groups that inhabit those areas [63,64].

Based on Euclidean distances to African and European PCA centroids (Figure 1c) we estimated a substantially higher European ancestry in the Forros than in the Principenses (13% vs. 3%). Using the same approach, we found Gulf of Guinea/Angolan ancestry ratios of 76%:24% in the Principenses and 66%:34% in the Forros (Supplementary Table S2). These ratios are in accordance with the low impact of Bantu lexicon in Lung’Ie when compared to Lungwa Santome, but do not reach statistical significance (Mann–Whitney *p* = 0.09). Ancestry estimates based on the frequency of ADMIXTURE components were highly correlated with the PC-based results (rho = 0.99; *p* < 10^−5^ for European ancestry estimates; rho = 0.81; *p* < 2 × 10^−4^ for Gulf of Guinea/Angolan estimates; Supplementary Table S2).

As the extreme divergence of the Angolares does not allow for an assessment of their ancestral proportions using PCA or ADMIXTURE, we additionally estimated Gulf of Guinea/Angolan ratios using an ad hoc approach based on the relative position of the three populations from São Tomé and Príncipe in the TVDxy/Neighbor-Joining network (Figure 2c). In agreement with the outstanding lexical Bantu contribution in Lunga Ngola, the Angolares display a lower Gulf of Guinea/Angolan ratio (58%:42%) than the other two groups (Principenses: 69%:31%; Forros: 64%:36%). 

### 3.2. Genetic Diversity

**Figure 3:**
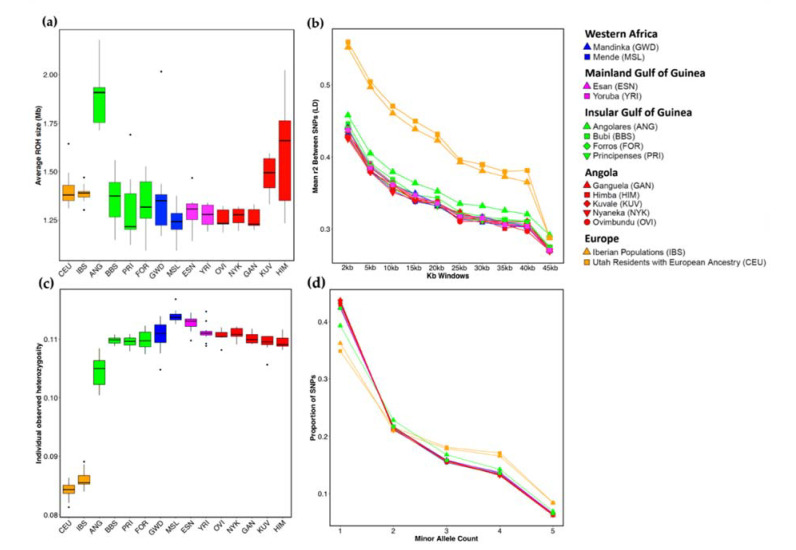
Summary statistics of genetic diversity. (**a**) Boxplots representing the individual variation in the average size of runs of homozygosity (ROH), defined as the ratio between the total length of ROH (sROH) and the number of ROH (nROH). (**b**) Linkage disequilibrium (LD) decay with physical distance. (**c**) Boxplots representing the variation in individual observed heterozygosity per locus (Ho). (**d**) Site frequency spectra (SFS). For (**b**,**d**), populations were randomly downsampled (without replacement) to the smallest sample size, and the average over replicates is reported.

Despite their mixed ancestry, the Angolares display substantially lower levels of genetic diversity than any other African population in our dataset. The patterns of ROH presented in Supplementary Figure S5 provide a remarkable illustration of this homogeneity. Both the number of ROH (nROH) and the average total length in ROH (sROH) of the Angolares are only surpassed by the European populations, who have experienced a bottleneck during the Out-of-Africa migration [65]. Consideration of the average ROH size (sROH/nROH) further shows that the Angolares have unusually long ROH, even when compared with the Europeans, as expected for populations with recent inbreeding [66,67] (Figure 3a). Interestingly, a similar, albeit less pronounced, trend is observed in the Himba and Kuvale from southwestern Angola, who have a well-documented preference for cross first-cousin marriages between a man and his father’s sister’s daughter [68]. Consistent with these observations, the Angolares, the Himba and the Kuvale stand apart from the other populations especially for longer ROH categories, measuring more than 2 Mb (Supplementary Figure S5d). A further indication of recent inbreeding is provided by a plot of sROH vs. nROH, showing that ROH sizes in the Angolares are longer than expected from their number of ROH, on the basis of the best-fitting line for sROH vs. nROH in outbred populations from mainland Africa (Supplementary Figure S6). However, the signals of inbreeding revealed by the ROH analyses are coupled with negative *Fis* values that are similar to other populations, and no significant differences in mating patterns could be captured using this statistic (Supplementary Figure S7). 

Additional characterization of other aspects of genetic diversity shows that the Angolares have higher levels of LD (Figure 3b, Supplementary Figure S8), lower observed per locus heterozygosities (Ho; Figure 3c) and lower proportions of singletons in the site spectrum (SFS; Figure 3d)) than the other African populations. All these summary statistics are strongly intercorrelated (Supplementary Figure S9) and suggest that the low levels of genetic diversity observed among the Angolares were caused by a comparatively small effective population size.

### 3.3. Reanalyzing Previously Generated Uniparental Data

Previously, we found that a single Y-chromosome microsatellite haplotype reached an unusually high frequency (15/25) in the Angolares, who otherwise retained a small number of equally frequent, molecularly divergent mtDNA lineages. This pattern contrasted with the high variability detected for the two uniparental markers in a sample of linguistically uncharacterized non-Angolar residents of São Tomé [22]. Using newly generated data, we now found that levels of mtDNA and Y-chromosome variability in Príncipe are similar to the non-Angolar sample from São Tomé (Supplementary Figure S10; Supplementary Tables S3–S6).

Moreover, we reassessed the provenance of the most common Angolar patrilineage by investigating its matching profile, using publicly available data from the Y- Chromosome Haplotype Reference Database (yhrd.org). We found that matches with Angola represent 39% (7/18) of all matches with African populations, although the Angolan sample size accounts for only 12% (309/2679) of the total sample size of populations in which at least one match was observed (Supplementary Figure S11; Supplementary Table S7). These results suggest that the most frequent Angolar Y-chromosome lineage is likely to have originated in Angola.

**Figure 4:**
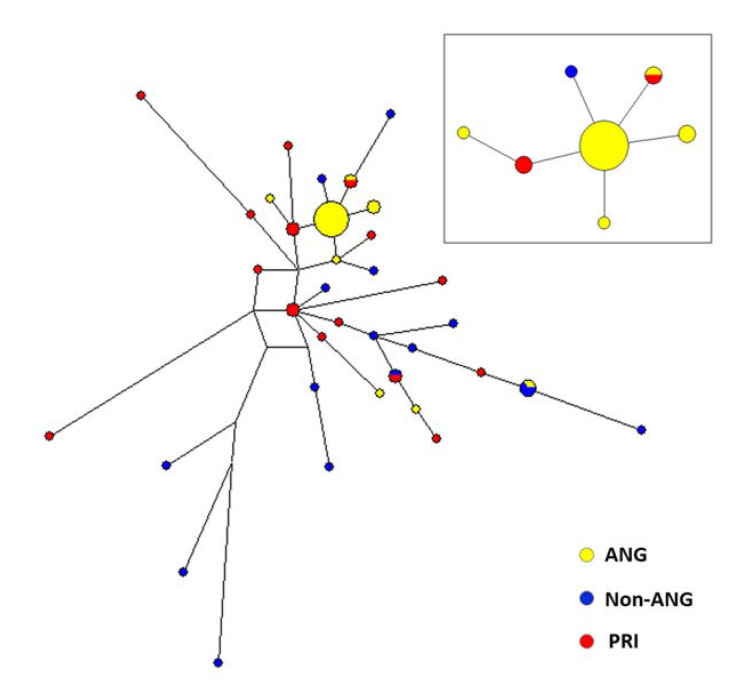
Network representing the haplotype variation in African-derived Y-chromosomes from São Tomé and Príncipe. Haplotypes were defined by using 10 microsatellite loci (DYS19, DYS389I, DYS389II, DYS390, DYS391, DYS392, DYS393, DYS437, DYS438, DYS439). Locus DYS385 was excluded from the network because it is duplicated. The inset shows the descent cluster centered around the most frequent haplotype in the Angolares. Haplotypes from the Angolares (ANG) and from a sample of linguistically uncharacterized non-Angolar residents of São Tomé (Non-ANG) were previously reported [22]. The newly generated data on the Y-chromosome haplotypes from Príncipe (PRI) are shown in Table S4. Circles represent haplotypes, area is proportional to frequency, and colors represent populations. Lines represent microsatellite mutational differences.

We also attempted to estimate the time to the most recent common ancestor (TMRCA) of this lineage. By using previously defined criteria [61], we first delimited a descent cluster of close mutational neighbors that likely derived from the most frequent (ancestral) haplotype (Figure 4). Then, we calculated the time necessary to generate the descent cluster through mutation accumulation, based on the rho statistic [58,59]. Our estimate suggests a TMRCA of ~500 years (95% confidence interval limits ~62–940 years), in broad agreement with historical records indicating that the first slaves from Congo-Angola arrived at São Tomé around 1520.

## 4. Discussion

The emergence of new ethnic identities defined by distinct cultural and linguistic traits is one of the most remarkable outcomes of the forced displacement of millions of Africans during the Atlantic Slave Trade. In contrast to other instances of European conquest where local societies were subjected to colonial rule, the plantation complex that was installed in the New World, especially in the Caribbean, relied on the mass replacement of indigenous groups with Africans of different geographical origins [1]. Although the coercive amalgamation of people from diverse backgrounds became the defining characteristic of the rapidly developing creole societies, enslavement and forced migration were also met with staunch resistance. Escapes (marronage) and rebellions were a frequent occurrence in all slave societies and eventually led to the creation of independent maroon communities surrounding the plantations [69].

Located right off the African coast, São Tomé and Príncipe anticipated some of these defining characteristics of Caribbean creole societies both in time and space [70]. While members of the modern Forro (São Tomé) and Principense (Príncipe) communities are generally understood to be the descendants of plantation slaves recruited in the Gulf of Guinea and Congo-Angola, the Angolares from São Tomé may represent the oldest maroon society formed during the Atlantic Slave Trade [8]. However, the specific conditions under which this self-governed group emerged remained to be fully elucidated.

By using 15 autosomal microsatellite polymorphisms together with mtDNA partial sequencing and Y-chromosome microsatellites, we have previously detected an unusually strong signal of genetic differentiation between the Angolares and a heterogeneous sample including non-Angolares inhabitants of the island of São Tomé [22]. However, the extreme differentiation of the Angolares, the paucity of comparative data and the low number of genetic markers analyzed did not allow us to recover the historical relationships between this community and other groups from São Tomé and Príncipe and from the African mainland.

Here, we used newly generated genome-wide data to show that when the impact of genetic differentiation is reduced, the Angolares display mixed contributions from the Gulf of Guinea and Angola (58%:42%) and are genetically closer to the Forros and the Principenses than to any other African population (Figure 2). At the same time, we confirm and extend the evidence indicating that the gene pool of the Angolares is unusually homogeneous, as shown by several summary statistics capturing different but related aspects of genetic diversity (LD; SFS; and Ho), including long ROH suggestive of substantial inbreeding (Figure 3; Supplementary Figures S5–S9). This combination of features rules out the possibility that the Angolares originated from a specific region of Africa, as assumed by the frequently cited hypothesis according to which they descend from survivors of the wreck of a slave ship carrying captives from Angola [4,8]. Alternatively, it is likely that the Angolares constitute an admixed isolate that was founded through fusion of a small number of slaves with different geographical backgrounds, and they subsequently experienced high levels of genetic drift and extensive isolation in the context of marronage.

Linguistic evidence offers additional insights into this scenario. The creoles spoken by the Forros (Lungwa Santome) and Angolares (Lunga Ngola) differ in the way in which Bantu-derived features are ingrained into different linguistic domains [11,12]. While the Kikongo influence in Lungwa Santome mostly consists of non-core lexical items, Kimbundu features in Lunga Ngola are found in multiple subsets of the language, including core vocabulary and phonology. This qualitatively different impact of the Bantu adstrate in Lunga Ngola suggests that the Angolares resulted from a union between creole-speaking slaves escaping from the plantations and recently arrived Kimbundu-speaking slaves from Angola, who had a considerable influence on the formation of the new language.

An important clue about this process is provided by the finding that the Angolares display a very high frequency of a single Y-chromosome microsatellite haplotype with a likely Angolan origin [22] (Figure 4; Supplementary Figure S11). Similarly to well-known examples of social selection [61,71], this pattern suggests that the founding Angolar population was dominated by a high-ranking Angolan male, or a small group of related males, who achieved greater reproductive success than other men and passed their elevated social status to their male descendants, favoring the rapid expansion of a single patriline.

A high status of prominent Angolan men provides the socio-linguistic context that could easily explain the emergence of Lunga Ngola through the partial relexification of a pre-existing creole under the influence of Kimbundu-speaking leaders. Moreover, the role of headmen, also known as “captains”, in the Angolares community has long been attested by historical sources [4,8]. As in many other maroon societies, transmissible male dominance is likely to have been favored by a highly centralized political organization under the strong authority of headmen who had the prerogative of polygyny and could transmit this mating advantage to their offspring [69]. Historical records additionally report episodes of women abduction from the farms [4,8], suggesting that the maroon communities experienced shortages of females that may have been caused or exacerbated by polygyny. Only after the death of their last captain, Simão Andreza, in the beginning of the 20th century, did the long history of chieftainship among the Angolares come to an end [4,8].

The effects of cultural transmission of male social dominance can be illustrated with a simple deterministic model, where the favored cultural phenotype (higher status) is associated with higher rates of polygyny among individuals inheriting the Y-chromosome from dominant males [72,73]. For example, assuming that dominant men represent 5% of the male population, a mating success three times higher than that of other males would be necessary for the current frequency of the Angolar descent cluster to be reached during the ~500 years corresponding to its estimated TMRCA (Supplementary Figure S12).

Even when driven solely by males, social selection is expected to have a strong whole-genome impact. In their pioneer studies on the genetic structure of Native American tribes, James Neel and his colleagues [74,75] have shown that the transmission of polygyny among high-ranking men could increase inbreeding to higher levels than expected by systematic marriage among relatives [4,8]. Our observation that the Angolares display higher amounts of inbreeding than southwestern Angolan groups, such as the Himba and Kuvale, who favor cross-cousin marriage, is congruent with those findings (Figure 3a; Supplementary Figures S5 and S6). Other known consequences of cultural transmission of fitness, including a sharp reduction in effective population size (Ne) and a strong increase in allelic association [76], could explain the low genetic diversity and increased LD of the Angolares (Figure 3). Together with strong isolation, this reduction in Ne probably led to the group’s unusual genetic divergence from the other populations of São Tomé and Príncipe.

While the observed patterns of Y-chromosome and genome-wide variation of the Angolares can also be explained by neutral demographic factors such as strong bottlenecks and founder effects, neutrality and social selection are of course not mutually exclusive. Therefore, further work is needed to clarify the roles played by these evolutionary factors in shaping the genetic and non-genetic dimensions of human diversity in São Tomé and Príncipe. Our genome-wide results provide the empirical framework for these analyses.

## Data Availability

Sequence data of the individuals analyzed in the present work have been deposited in the European Nucleotide Archive (ENA) repository under the accession number PRJEB44717.

## Supplementary Materials

The following are available online at https://www.mdpi.com/article/10.3390/genes12060833/s1, Figure S1: Pairwise Fst distances between populations; Figure S2: Haplotype-based Principal Component Analysis; Figure S3: ADMIXTURE analysis; Figure S4: Dendrogram displaying genetic relationships between studied individuals; Figure S5: Individual variation in measures of runs of homozygosity (ROH); Figure S6: Comparison between number of ROH (nROH) and total length of ROH (sROH); Figure S7: Variation in individual F_is_ values; Figure S8: Variation in average pairwise linkage disequilibrium (LD); Figure S9: Pairwise correlations between summary statistics of genetic diversity; Figure S10: mtDNA and Y-chromosome networks; Figure S11: Matching analysis; Figure S12: Frequency change in Y-chromosome descent cluster; Table S1: Geographic location, language affiliation and sample size of populations used in this study; Table S2: Ancestry estimates; Table S3: mtDNA variation in Príncipe; Table S4: Y-chromosome variation in Príncipe; Table S5: mtDNA diversity statistics; Table S6: Y-chromosome diversity statistics; Table S7: Y-chromosome haplotype sharing results.
