# Supplementary material for: The Genes of Freedom: Genome-Wide Insights into Marronage, Admixture and Ethnogenesis in the Gulf of Guinea
Source: Genes (Basel). 2021 May 28;12(6):833. doi: 10.3390/genes12060833 (PMC8229774; doi:10.3390/genes12060833)
Supplement: Supplementary file 1 [file genes-12-00833-s001.zip › Supplementary_Figures.pdf]

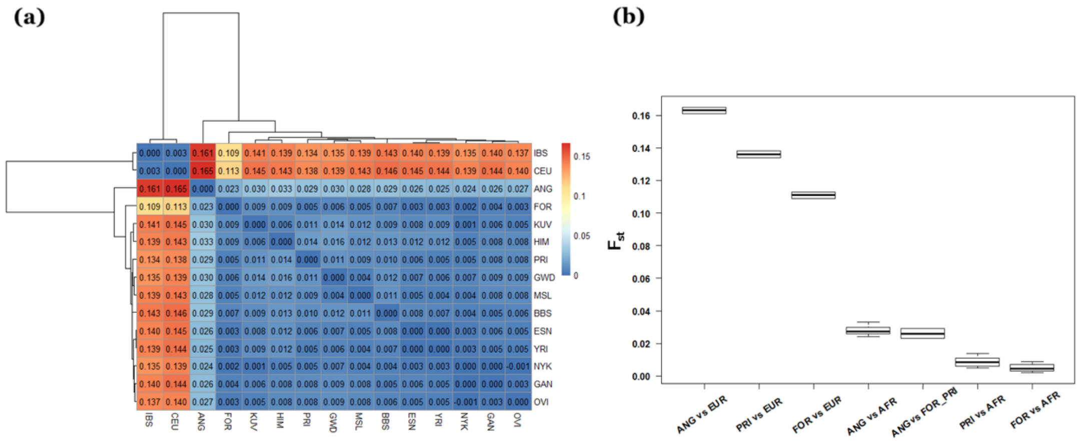

Figure S1. (a) Matrix of pairwise Fst genetic distances between populations; **(b)** Boxplots displaying the distributions of Fst genetic distances between different pairwise combinations of populations. ANG=Angolares; EUR=Europeans; PRI=Principenses; FOR=Forros; AFR=Africans (Mandinka, Mende, Esan, Yoruba, Bubi, Ganguela, Himba, Kuvale, Nyaneka, Ovimbundu).

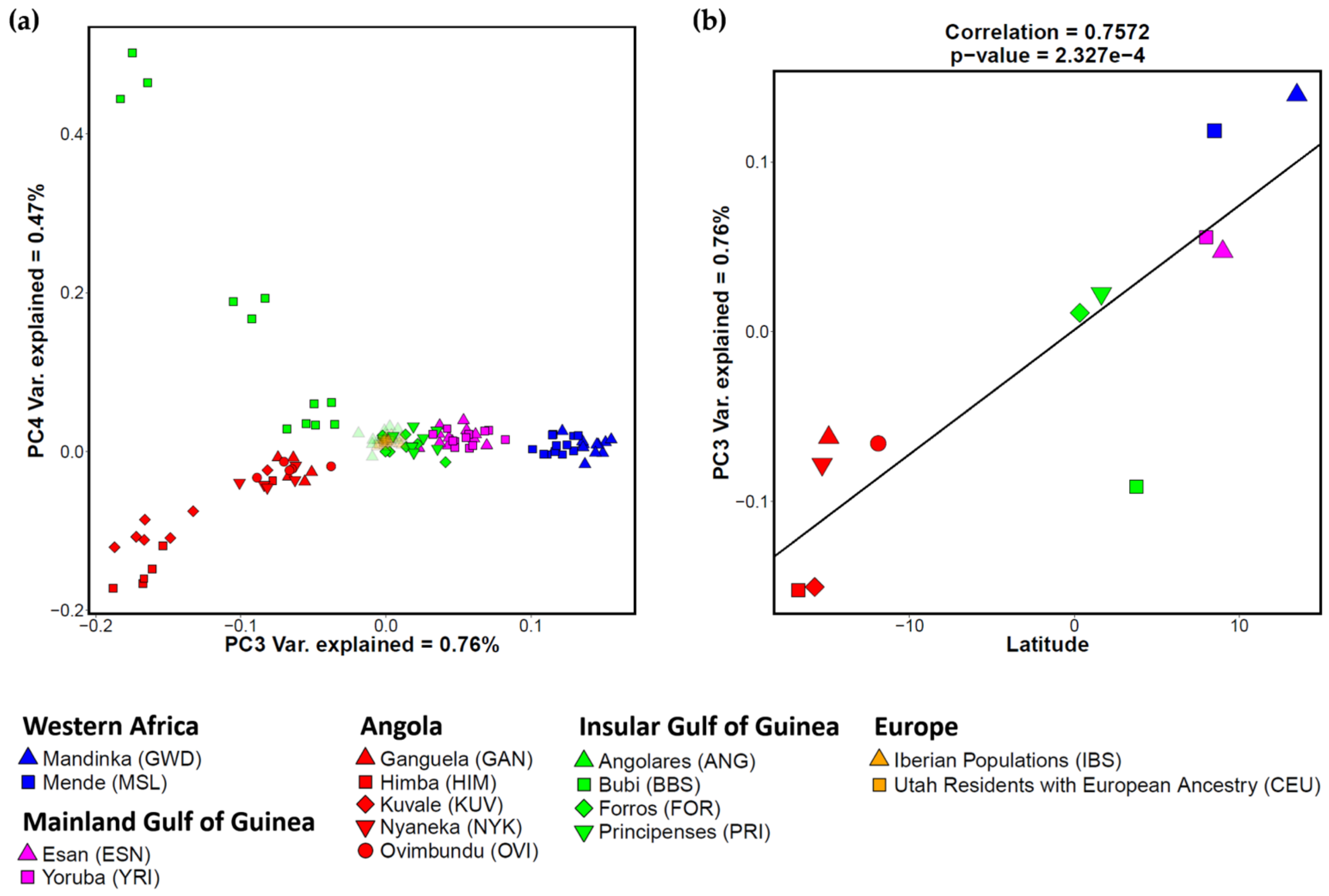

Figure S2. (**a-b**) Haplotype based Principal Component analysis; **(a)** PC3 and PC4 plot; **(c)** Correlation between average PC3 scores and latitude.

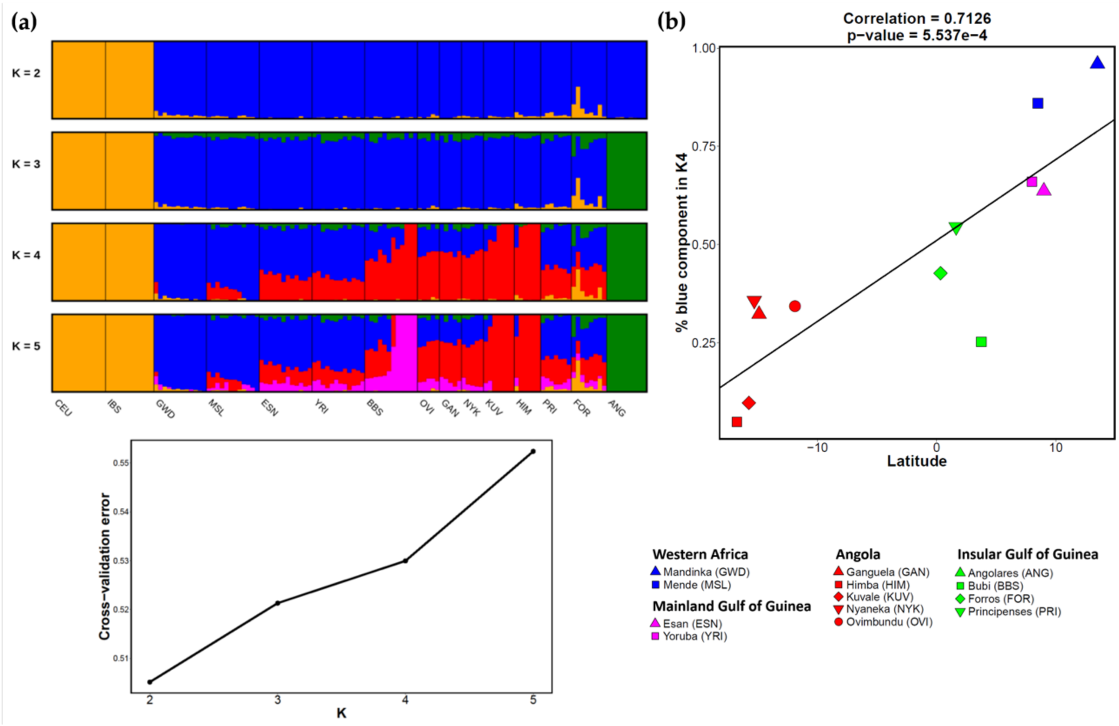

Figure S3. (**a**) ADMIXTURE analysis from K=2 to K=5 (CV values are displayed in the inset). **(b)** Correlation between the average proportions of the blue component (K=4) and latitude.

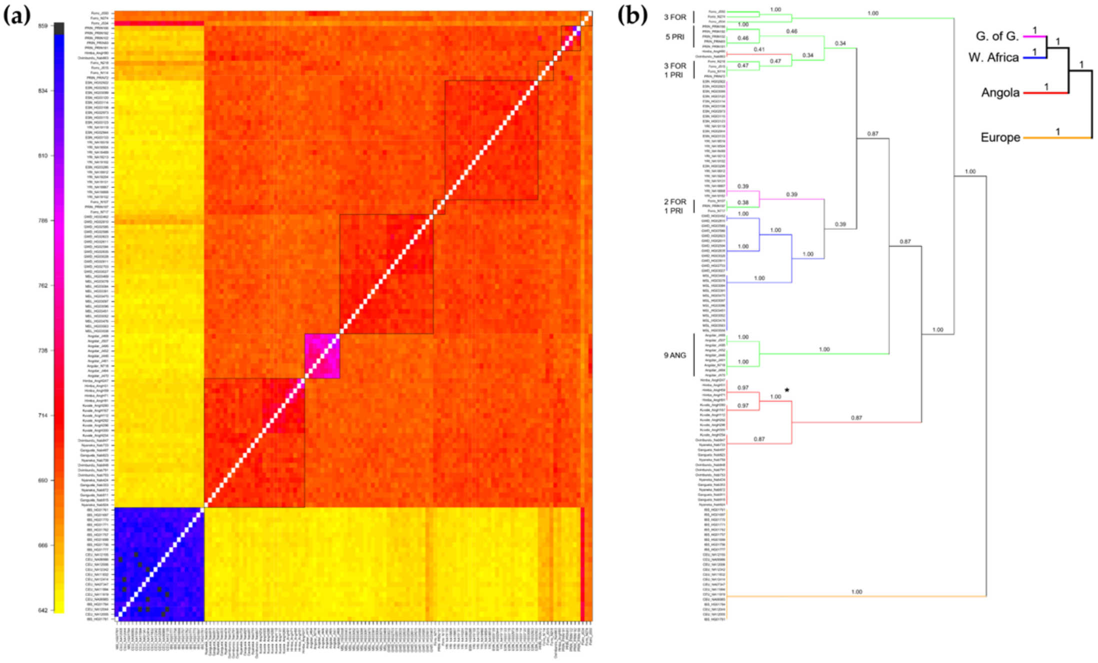

Figure S4. (**a**) CHROMOPAINTER/fineSTRUCTURE coancestry matrix based on the number of haplotype segments (chromosome chunks) shared between individuals from the studied populations. The scale of chunk counts is shown to the left of the matrix. **(b)** Dendrogram displaying the relationships between individuals based on the coancestry matrix from panel (a). The star shows a cluster encompassing the pastoralist Himba and Kuvale, which were excluded from the set of Angolan parental populations in admixture estimates. The appended tree summarizes the branching pattern obtained when the coancestry matrix is calculated without the populations from São Tomé and Príncipe. W. Africa= West Africa; G. of G. = Gulf of Guinea.

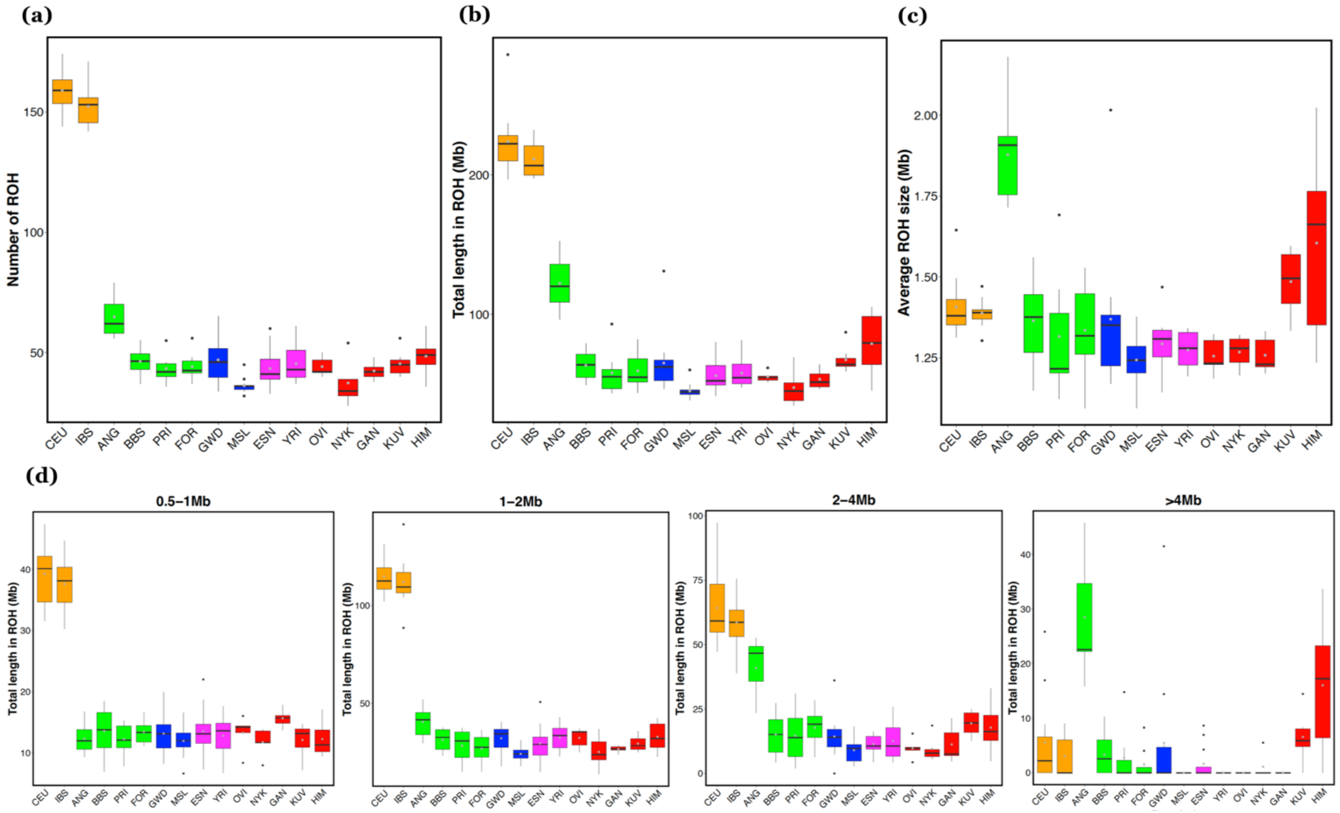

Figure S5. Boxplots representing the individual variation in measures of runs of homozygosity (ROH) in the studied populations. **(a)** Number of ROH (nROH); **(b)** Total length in ROH (sROH); **(c)** Average ROH size (sROH/nROH) (**d**) Total length in ROH over four classes of length categories: 0.5-1Mb; 1-2Mb; 2-4Mb; >4Mb

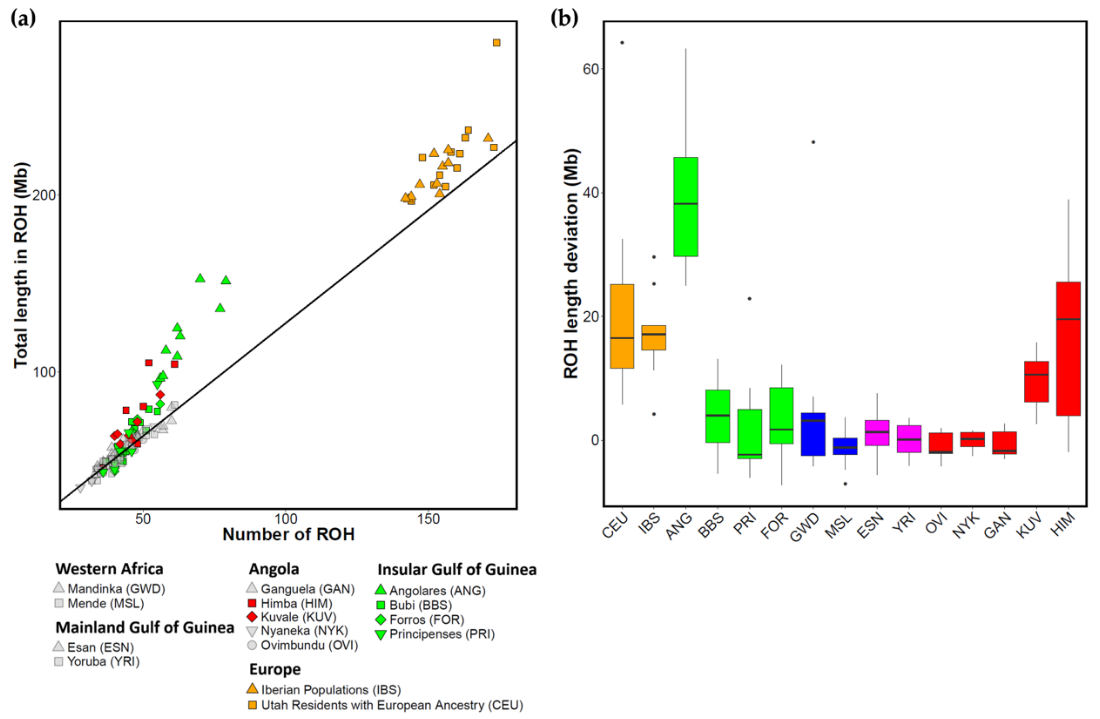

Figure S6. Comparison between the number of ROH (nROH) and the total length of ROH (sROH). **(a)** Plot of nROH versus sROH showing the line of best fit for outbred African populations (here displayed in grey); **(b)** Variation in the differences between the observed sROH of each individual and the sROH to be expected from its nROH, as calculated from the best fitting line in panel (a).

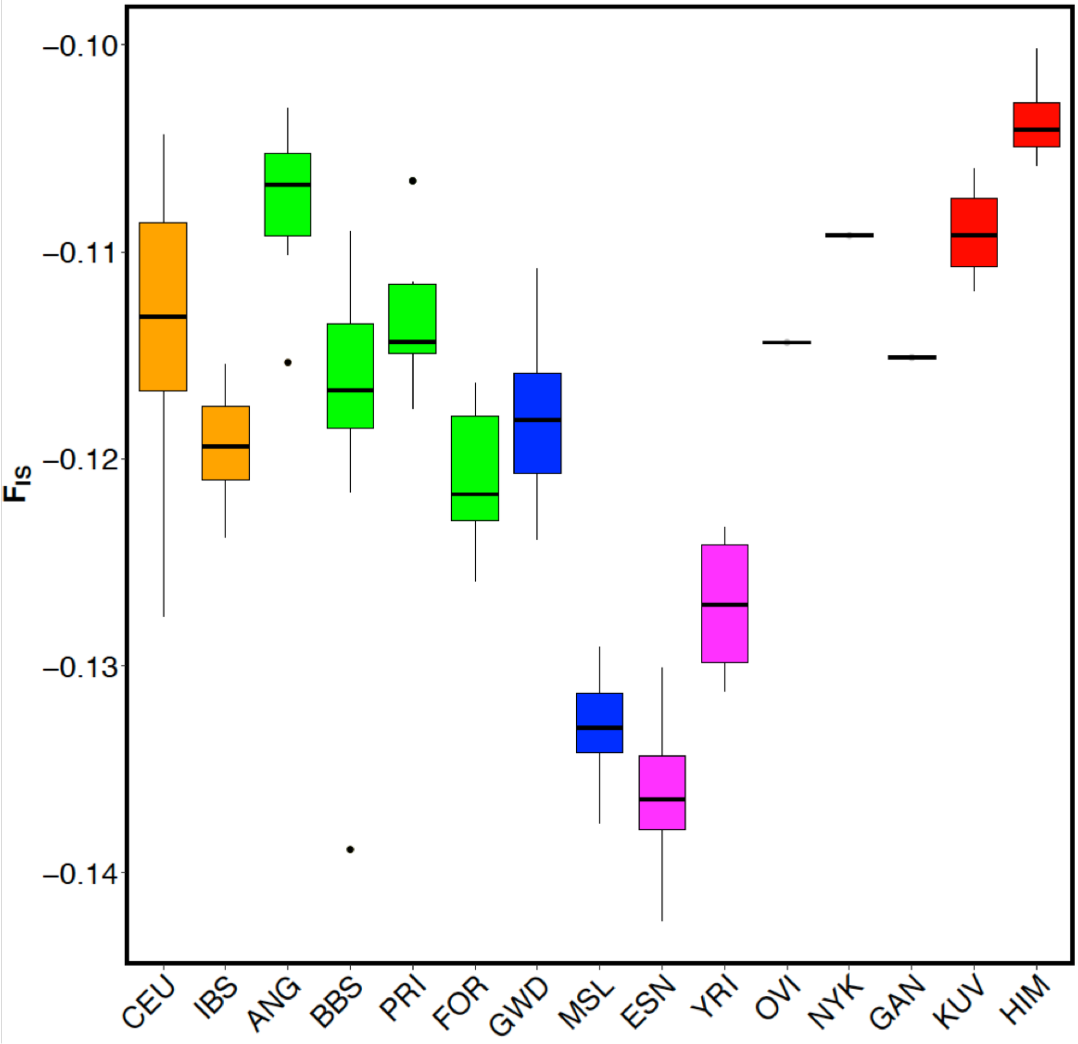

Figure S7. Variation in the averages of individual F_{is} values over downsampling replicates obtained for each population. Populations were randomly downsampled (without replacement) to the smallest sample size (populations with smallest sample size - OVI, NYK, GAN - have no replicates).

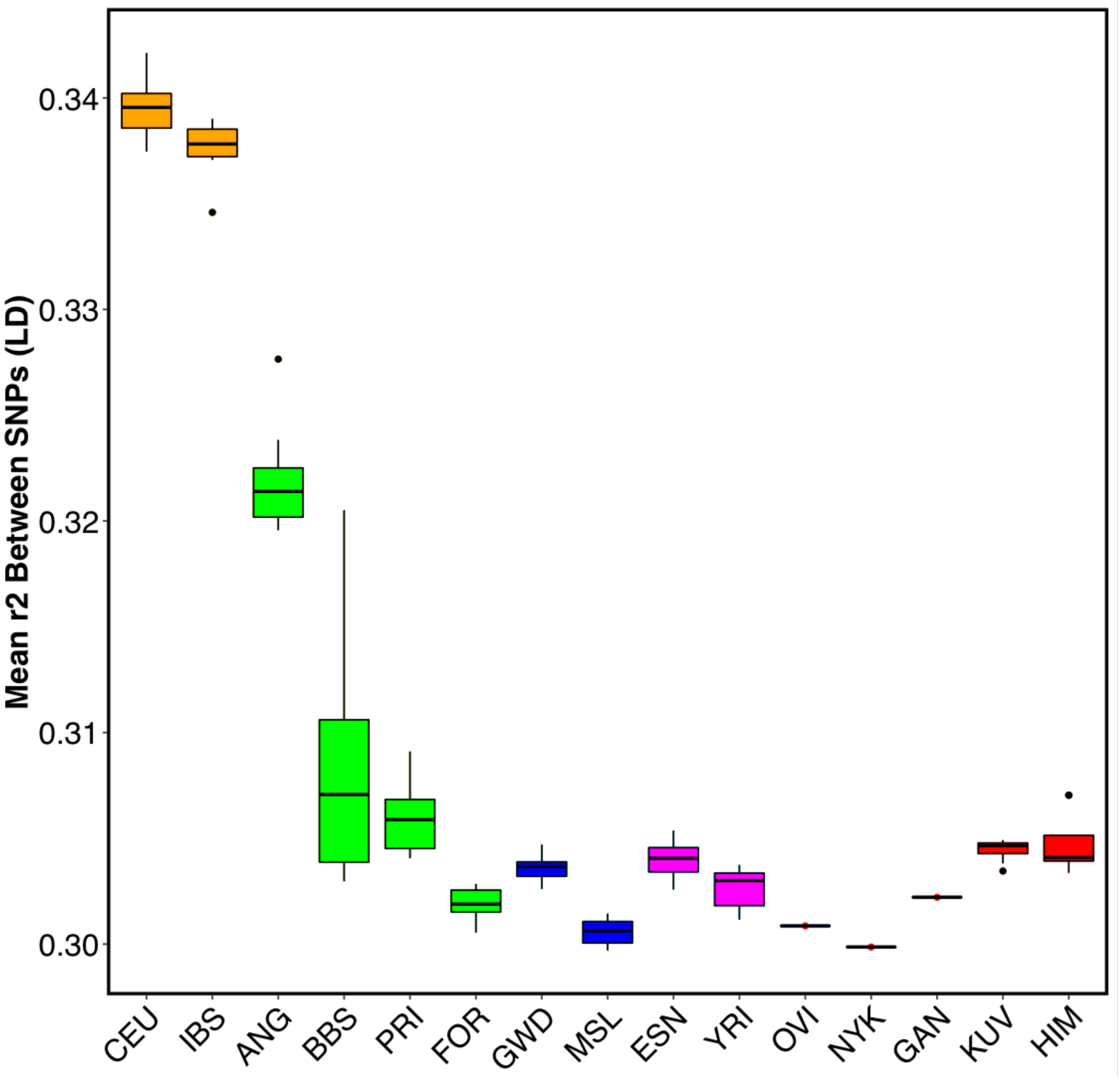

Figure S8. Boxplots representing the variation in average pairwise linkage disequilibrium (LD). We report the variation in the averages over downsampling replicates (populations with smallest sample size – OVI, NYK, GAN – have no replicates).

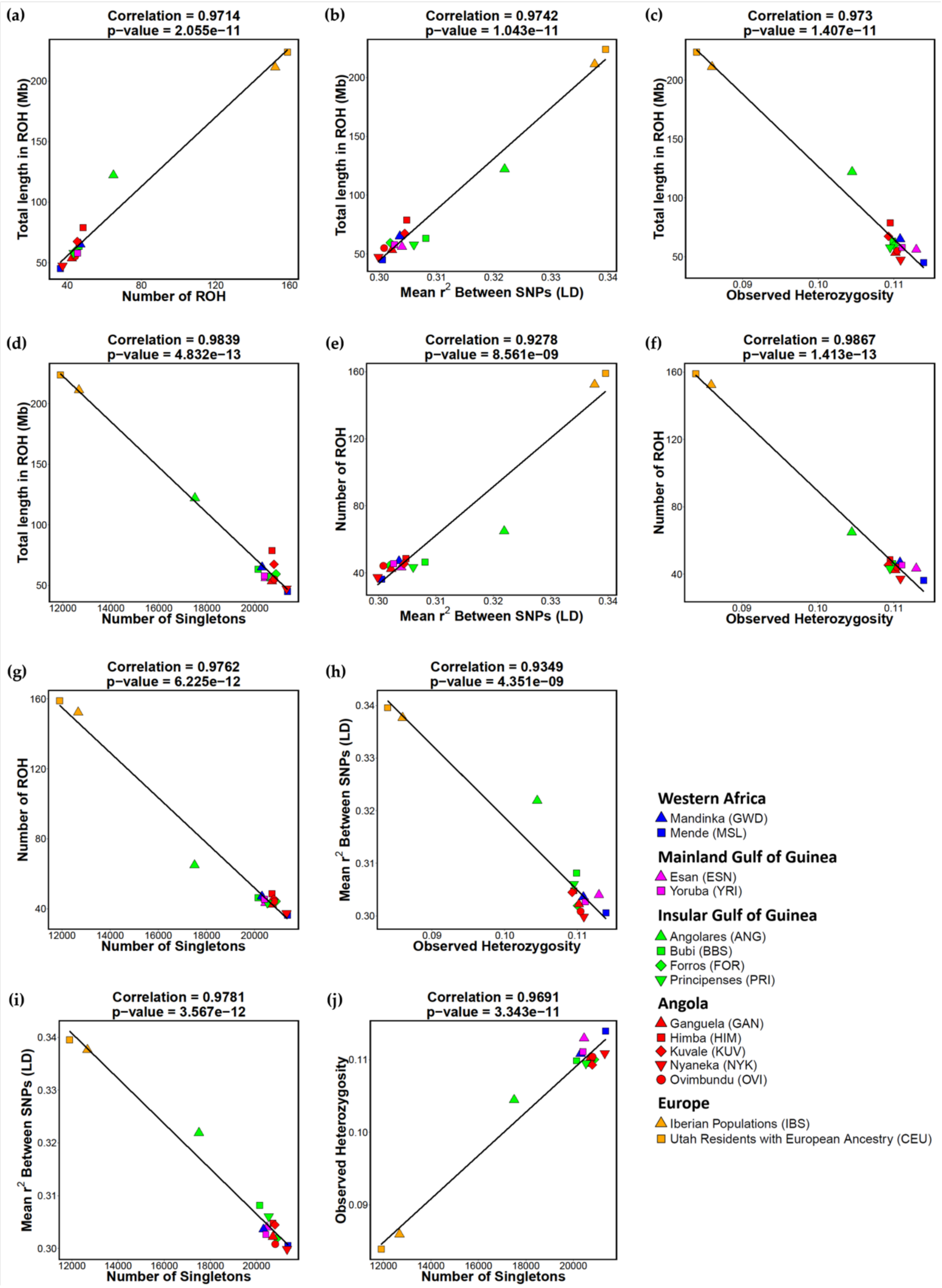

Figure S9. Pairwise correlations between summary statistics of genetic diversity.

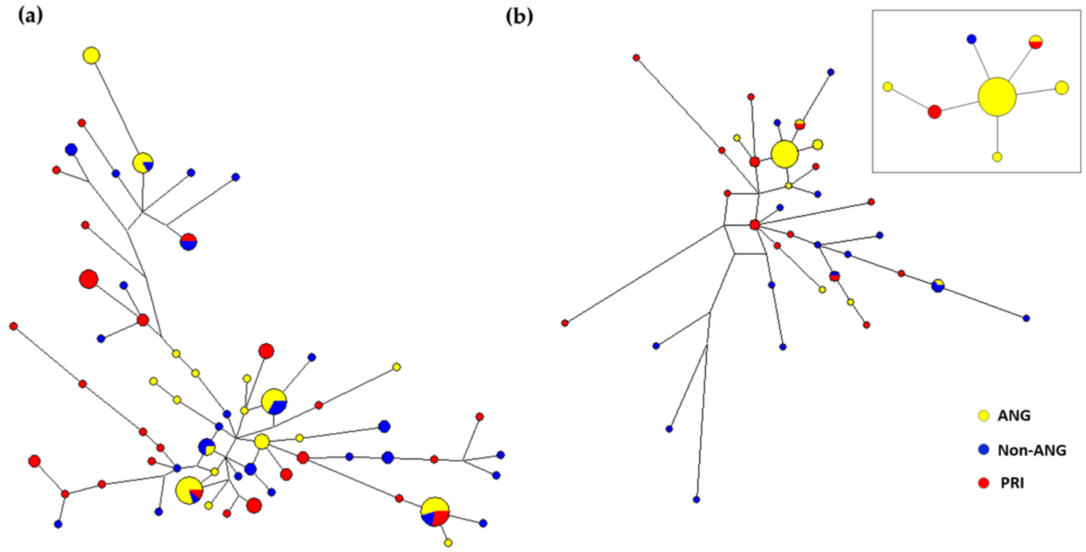

Figure S10. (a) Network representing mtDNA HVR-I sequence variation in São Tomé e Príncipe. **(b)** Network representing haplotype variation in African-derived Y-chromosomes from São Tomé and Príncipe. The inset shows the descent cluster centered around the most frequent haplotype in the Angolares. MtDNA HVR-I and Y-chromosome haplotypes from the Angolares (ANG) and from a sample of linguistically uncharacterized non-Angolar residents of São Tomé (Non-ANG) were previously reported [22]. The newly generated data on the mtDNA and Y-chromosome from Príncipe (PRI) are shown in Supplementary Tables S3 and S4, respectively. Circles represent haplotypes, area is proportional to frequency, and colors represent populations. Lines represent microsatellite mutational differences.

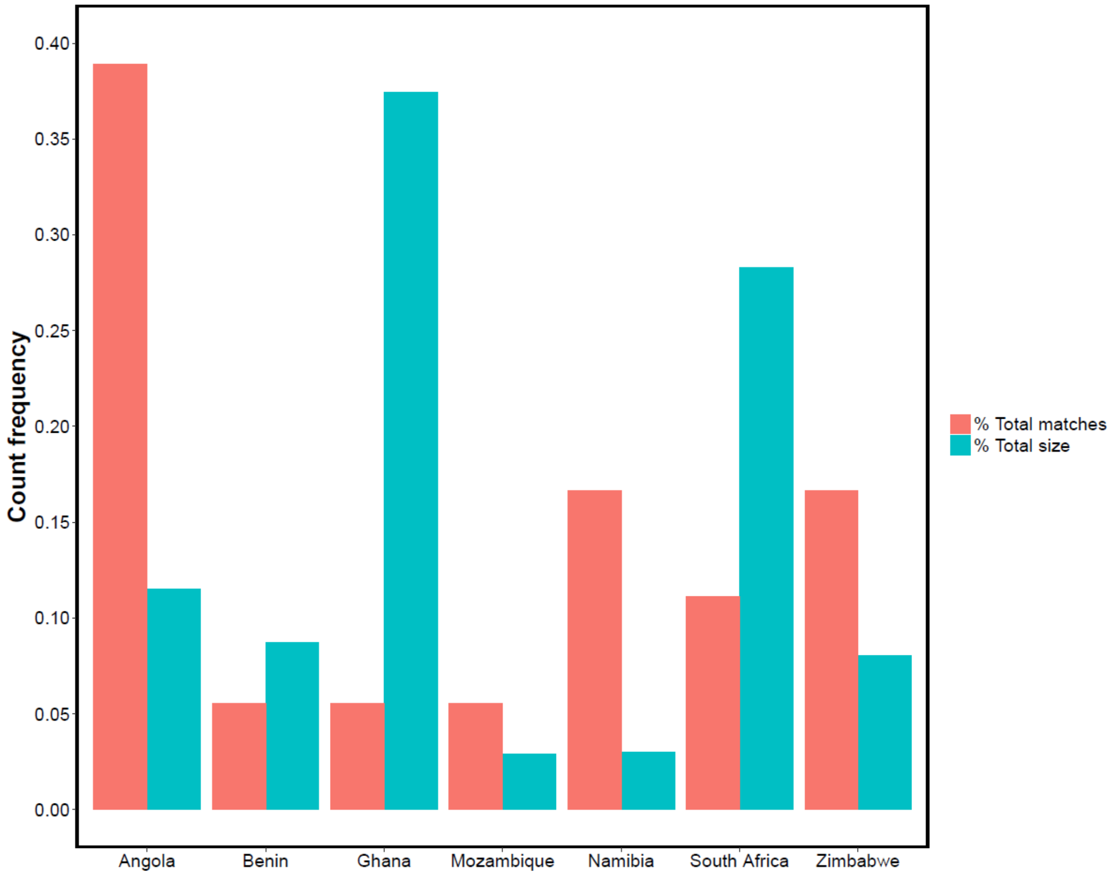

Figure S11. Matching patterns between the most frequent Y-chromosome microsatellite haplotype in the Angolares and Y-chromosomes from continental African populations. "% Total matches" is the ratio between the number of exact matches found in a given population and the total number of exact matches with African populations. "% Total size" is the ratio between the sample size of that population and the total sample size of populations in which at least one exact match was observed. Matching analysis was done by using data available in the Y-STR Haplotype Reference Database (YHRD). Additional information on matching profiles is given in Supplementary Table S7.

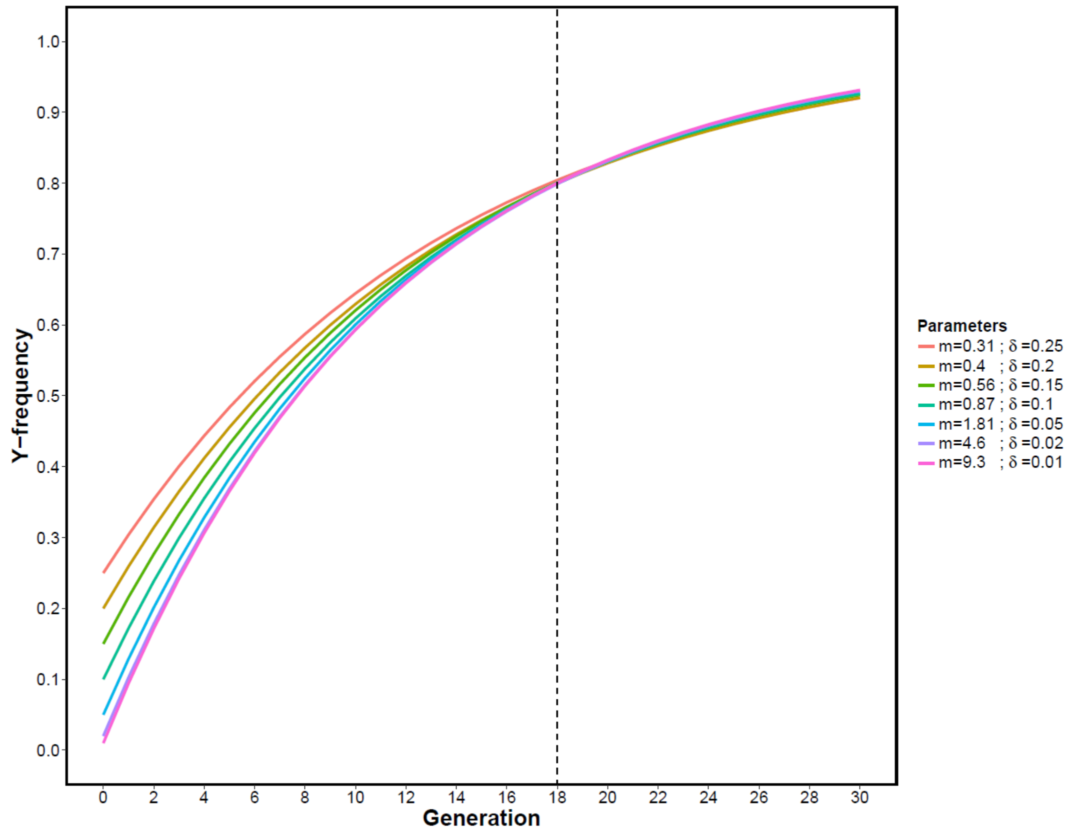

Figure S12. Change in frequency of a Y-chromosome descent cluster associated with dominant males, whose mating success is higher than that of nondominant males. In this simple deterministic model, m measures the added mating success (1+m) of dominant males bearing the favored descent cluster and δ measures the percentage of the male offspring of dominant men that become dominant themselves [72,73]. For simplicity, this percentage is kept constant and equal to the initial frequency of the descent cluster. We show different combinations of m and δ that lead to the present frequency of the favored descent cluster in the Angolares in 18 generations (the estimated descent cluster TMRCA for generation times between 25 and 30 years). Note that the frequency of the entire descent cluster and not just the frequency of its most frequent haplotype is taken into consideration.
